# Dezocine, An Opioid Analgesic, Exerts Antitumor Effects in Triple-Negative Breast Cancer by Targeting Nicotinamide Phosphoribosyltransferase

**DOI:** 10.3389/fphar.2021.600296

**Published:** 2021-04-12

**Authors:** Chenyang Xue, Wei Chen, Aiwu Yuan, Cheng Chen, Shuaihu Li, Kai Chen, Yang Zhao, Tian Xiao, Genze Shao, Yongdong Zou, Duo Zheng

**Affiliations:** ^1^ Guangdong Provincial Key Laboratory of Regional Immunity and Diseases, Shenzhen University International Cancer Center, Department of Cell Biology and Genetics, School of Medicine, School of Pharmaceutical Sciences, Health Science Center, College of Life Sciences and Oceanography, Shenzhen University, Shenzhen, China; ^2^ Department of Anesthesiology, Longgang District Maternity & Child Healthcare Hospital of Shenzhen City, Shenzhen, China; ^3^ College of Chemistry and Chemical Engineering, Central South University, Changsha, China; ^4^ Department of Cell Biology, School of Basic Medical Sciences, Peking University, Beijing, China

**Keywords:** triple-negative breast cancer, dezocine, opioid, NAMPT, proliferation, metastasis

## Abstract

Opioids are a potential adjuvant treatment for certain cancers; while they are primarily used to relieve chronic pain, these drugs may also affect cancer progression and recurrence. Dezocine is one opioid commonly used in China, but its effects on cancer cells are unknown. Here, we demonstrated the inhibitory effect of dezocine on triple-negative breast cancer (TNBC) cells, and determined the underlying molecular mechanism. We found that dezocine suppressed cell proliferation, migration and invasion, and induced apoptosis in TNBC cells. Xenograft models demonstrated the inhibitory effects of dezocine treatment on TNBC tumor growth *in vivo*. The anticancer effects of dezocine were independent of opioid receptors, which are not highly expressed by normal breast or breast cancer tissues. A pull-down assay and LC-MS/MS analysis indicated that dezocine directly targets NAMPT: computer modeling verified that the free energy of dezocine kinetically bound into the pocket of NAMPT was −17.4 kcal/mol. Consequently, dezocine treatment inhibited NAMPT enzyme activity, resulting in cellular NAD abolishment. We confirmed the dezocine-induced inhibition of cell proliferation by both NAMPT knockdown and upon treatment with the inhibitor FK866. Our results suggest that both dezocine and NAMPT might represent novel therapeutic targets for TNBC.

## Introduction

Breast cancer is the most common malignancy suffered by women, accounting for 30% of all diagnosed cases (Siegel et al., 2019). Advances in disease treatment and management have improved survival rates, but breast cancer patients still experience the second highest mortality rate of all female cancer patients. However, mortality rate differs depending on breast cancer subtype, with some being more aggressive and difficult to treat than others. One example is triple negative breast cancer (TNBC), which accounts for 15% of all diagnosed breast carcinomas and is characterized by a lack of estrogen receptor (ER), progesterone receptor (PR), and human epidermal growth factor receptor 2 (HER2) expression (Waks and Winer, 2019). This means that traditional treatments that target these biomarkers are largely ineffective, and patients with TNBC typically experience an unfavorable prognosis. At present, TNBC is treated with standard chemotherapy combined with PARP inhibitors or DNA-targeting platinum drugs, such as carboplatin (Aggarwal et al., 2019). However, the heterogenous nature of the disease means that even these aggressive, combination treatment regimens are often ineffective, and novel treatment approaches are urgently required to improve prognosis for patients with TNBC.

For this reason, our group sought to investigate the potential of opioid drugs as an adjuvant treatment for TNBC. Opioids are widely used for relieving moderate to severe pain in the clinic, including chronic cancer pain, but previous studies have suggested that regional anesthesia and analgesia may also impact cancer progression and recurrence (Tedore, 2015; Forget et al., 2019; Wall et al., 2019). Furthermore, both agonistic and antagonistic opioid ligands have been found to affect cancer growth and development (Boland et al., 2014; Szczepaniak et al., 2020). For example, the mu opioid receptor (MOR) serves an important role in cancer progression by regulating angiogenesis, EMT, mTOR, Src and other signaling pathways (Singleton et al., 2015). MOR is highly expressed in human non-small cell lung cancer (NSCLC) tumor tissues, and the MOR agonist morphine increases Lewis lung carcinoma (LLC) cell proliferation, while MOR knockout mice or the opioid antagonist MNTX infusion attenuates LLC tumor growth and reduces lung metastasis (Mathew et al., 2011). In addition, increased MOR expression is associated with shorter progression-free survival (PFS) and overall survival (OS) in patients with metastatic prostate cancer (Zylla et al., 2013). Recent studies have also linked MOR overexpression to hepatocellular carcinoma (HCC) progression and poor prognosis in HCC patients, while MOR silencing is found to reduce HCC-associated tumorigenesis (Chen et al., 2019; Li et al., 2019). Morphine promotes triple negative breast cancer (TNBC) progression, angiogenesis and metastasis in xenograft mouse models and *in vitro* studies (Bimonte et al., 2015; Liu et al., 2020), but the effect of morphine in published literature remains controversial (Bimonte et al., 2013). Another opioid analgesic, dezocine, is a MOR and kappa receptor (KOR) mixed agonist-antagonist, with a stronger affinity to MOR (Wang et al., 2018). It also acts as a norepinephrine and serotonin reuptake inhibitor via the norepinephrine transporter (NET) and serotonin transporter (SERT) (Liu et al., 2014). It is widely used in China, particularly for the relief of chronic cancer pain. The link to MOR suggests that dezocine treatment may also affect cancer progression and metastasis; however, the effect of dezocine on cancer cells remains unknown.

In the present study, we found that dezocine inhibited cell growth, induced apoptosis, and suppressed metastasis in TNBC cell lines. Furthermore, we determined that dezocine exerted these anticancer effects by directly targeting nicotinamide phosphoribosyltransferase (NAMPT), which triggered the downregulation of NAMPT enzyme activity and NAD levels. Importantly, Xenograft models indicated the inhibitory effects of dezocine *in vivo*. These findings suggest that dezocine may have potential as a novel adjuvant treatment to inhibit TNBC progression.

## Materials and Methods

### Cell Lines and Reagents

SH-SY5Y and U937 cell lines were obtained from the Beijing Stem Cell Bank, Chinese Academy of Sciences (Beijing, China). All other cell lines were purchased from the American Type Culture Collection (Manassas, VA, United States). MDA-MB-231, BT549, MDA-MB-468 and MCF7 cells were cultured in DMEM (HyClone; GE Healthcare, Chicago, IL, United States) containing 10% fetal bovine serum (FBS; PAN-Seratech GmbH, Aidenbach, Germany). HCC1937 and U937 cells were cultured in RPMI medium (HyClone; GE Healthcare) containing 10% FBS. MCF10A cells were cultured using the MEGM Bullet kit (Lonza Group, Basel, Switzerland) and 10 ng/ml cholera toxin. SH-SY5Y cells were cultured in DMEM/F12 (Gibco; Thermo Fisher Scientific, Inc., Waltham, MA, United States) containing 10% FBS. All cell lines were propagated at 37˚C with 95% humidity in a 5% CO_2_ incubator.

Dezocine was obtained from Yangzi River Pharmaceuticals Group (Taizhou, Jiangsu, China). FK866 and NMN were purchased from Selleck Chemicals LLC, Houston, TX, United States). Morphine was obtained from Northeast Pharmaceutical Group Shenyang No.1 Pharmaceutical Co. Ltd (Shenyang, Liaoning, China). Morphine, dezocine and NMN were dissolved in ddH_2_O. FK866 was dissolved in ethanol. All compounds were diluted in appropriate media for cell culture studies.

### Cell Viability Assay

Cell viability was measured using Cell Counting Kit-8 (CCK-8; Dalian Meliun Biotech Co., Ltd., Dalian, Liaoning, China) according to the manufacturer’s protocol. Cells were seeded in 96-well plates at a density of 5000 cells/well, and were incubated in a final volume of 100 μL culture medium per well. After 24 h, the cells were treated with dezocine or FK866 for 48 h and cell viability was tested via the addition of CCK-8 reagent. To determine the half maximal inhibitory concentration (IC50), cells were treated with different concentrations of dezocine (0–320 μg/ml) for 48 h, and cell viability was measured.

### Colony Formation and Proliferation Assays

Cells were seeded in 6-well plates at a density of 1000 cells/well and were incubated in a final volume of 2 ml/well culture medium containing different concentrations of dezocine (0, 10, 20 μg/ml). After 8–10 days incubation, the plates were washed with PBS and the cells were fixed with 100% methanol at 4˚C for 10 min. Then, cells were stained with 0.05% crystal violet for 10 min at room temperature, and washed twice with ddH_2_O.

DNA synthesis was detected in MDA-MB-231 and BT549 cells using the Cell-Light EdU Apollo488 *In Vitro* kit (Guangzhou RiboBio Co., Ltd., Guangzhou, Guangdong, China), according to the manufacturer’s protocol. Briefly, cells were seeded in a 96-well plate at a density of 5000 cells/well, and were incubated in a final volume of 200 ml/well culture medium containing different concentrations of dezocine (10–40 μg/ml) for 48 h. The cells were then stained with Apollo488 and Hoechst33342. Images in 5 fields for each sample were randomly captured, and EdU-positive and Hoechst 33,342-positive cells were counted using Image-Pro Plus 6.0 software (Media Cybernetics, lnc., Rockville, MD, United States)

### Cell Apoptosis Analysis

MDA-MB-231 and BT549 cells were seeded into 6-well plates. After 24 h, the cells were treated with dezocine at 0, 10, 20, 40 μg/ml for 48 h. The cells were then labeled with Annexin V and PI using the Annexin V-FITC Apoptosis Detection kit (BioVision, Inc., Milpitas, CA, United States) and were analyzed using FACSCalibur platform (BD Biosciences, Franklin Lakes, NJ, United States).

### Wound Healing Assays

Cells were seeded into 6-well plates, cultured until they reached 90% confluence, then starved in serum-free medium overnight. Following this, the cell monolayer was scratched using a sterile 200 μL tip and the plates were washed with PBS 2–3 times to remove floating cells. Then, DMEM containing 10% FBS and dezocine at 0, 10, 20, 40 μg/ml was added. Wound healing was monitored using an Olympus IX73 inverted microscope (Olympus Corporation, Tokyo, Japan) to capture photos of the migrating cells at 0, 24 and 48 h.

### Transwell Assays

A total of 5 × 10^5^ cells suspended in 200 μL serum-free medium were seeded in the upper chamber of 24-well Transwell inserts (8-μm pores, Corning; Thermo Fisher Scientific, Inc.) coated with or without Matrigel (BD Biosciences) for invasion and migration assays, respectively. The lower chamber contained 600 μL DMEM with 10% FBS. After incubation for 18 h, non-migrating cells on the upper side of the membrane were removed with a cotton swab, and the remaining cells were fixed and stained with 0.5% crystal violet. Images of 5 random fields were then captured using an Olympus IX73 inverted microscope (Olympus Corporation).

### Western Blot

MDA-MB-231 and BT549 cells cultured in 60 mm dishes were treated with dezocine at 0, 10, 20, 40 μg/ml for 48 h. The cells were collected with sample buffer containing protease inhibitors, and protein concentration was determined using a BCA Protein Assay kit (Thermo Fisher Scientific, Inc.). Protein samples were separated by SDS-PAGE, and then transferred onto polyvinylidene fluoride membranes. Then, the membranes were labeled with corresponding primary antibodies and horseradish peroxidase-conjugated secondary antibodies. Protein expression was then detected using the Pierce ECL western blot substrate (Thermo Fisher Scientific, Inc.). The antibodies used are listed in Supplementary Table S1.

### Total Ribonucleic Acid Isolation and RT-qPCR

Total RNA was extracted from the cultured cells and the concentration was measured with a Nanodrop spectrometer N1000 (Thermo Fisher Scientific, Inc.). The extracted RNA was then reverse-transcribed to cDNA using the Revert Aid First Strand cDNA Synthesis kit (Thermo Fisher Scientific, Inc.). The cDNA was then diluted 10-fold and prepared according to the SYBR Green Reagent specification (Takara Bio, Inc., Kusatsu, Japan). The protocol was as follows: 95˚C for 10 min; 40 cycles of 95˚C for 15 s and 60˚C for 30 s. mRNA expression of the target genes was analyzed using the 2-^ΔΔCq^ method. Each real-time PCR reaction was repeated three times and normalized to the internal reference *GAPDH*. The primers used are listed in Table 2.

### *In Vitro* Dezocine Pull Down Assay

2.5 mg Dezocine was incubated with Sepharose 4B beads (200 ml/g; GE Healthcare Life Sciences) in binding buffer (50 mM Tris, pH 7.5, 5 mM EDTA, 150 mM NaCl, 1 mM dithiothreitol, 0.01% Nonidet P-40, 4 μg/ml bovine serum albumin, 0.02 mM PMSF, 1X protease inhibitor mixture) with gentle rocking overnight at 4˚C to form dezocine-Sepharose 4B. The dezocine-Sepharose 4B beads were then washed three times with washing buffer (50 mM Tris, pH 7.5, 5 mM EDTA, 150 mM NaCl, 1 mM dithiothreitol, 0.01% Nonidet P-40, 0.02 mM PMSF). MDA-MB-231 cellular supernatant fraction (1 mg) was then incubated with 200 μL dezocine-Sepharose 4B or Sepharose 4B (as a negative control) in binding buffer. After incubation overnight at 4˚C with gentle rocking, the beads were washed five times with washing buffer, and the proteins bound to the beads were analyzed by LC-MS/MS and western blotting.

### Protein In-Gel Digestion

The proteins from dezocine pull down assay was separated and then Coomassie-stained bands on the polyacrylamide gel were excised and transferred into a 1.5 ml microcentrifuge tube, where they were rinsed twice with Mill-Q water. The spots were then de-stained with 100 μL 50 mM ammonium bicarbonate/acetonitrile (1:1, vol/vol) and incubated with occasional vortexing for 30 min, depending on the intensity of the staining. Then, the de-staining solution was discarded and the tubes were washed twice with 200 μL of Mill-Q water. Next, 400 μL 100% acetonitrile was added to dry the gel spots in a SpeedVac for 10 min. Finally, the gel spots were rehydrated in a minimal volume of sequencing-grade porcine trypsin or chrymotrypsin solution (Promega Corporation, Madison, WI, United States; 20 μg/ml in 25 mM NH_4_HCO_3_) and incubated at 37˚C overnight. Supernatant was transferred into a 200 μL microcentrifuge tube, and the gels were extracted with extraction buffer (67% acetonitrile containing 1% trifluoroacetic acid). The peptide extract and gel spot supernatant were combined and then completely dried in a SpeedVac.

### Mass Spectrometry and nanoLC-Electrospray Ionization-Mass Spectrometry Analysis

The lyophilized peptide was re-suspended in 2% acetonitrile containing 0.1% formic acid and then 4 μL aliquots was loaded into a ChromXP C18 (3 μm, 120 Å) trap column. Online chromatography separation was performed on the Ekspert nanoLC 415 system (SCIEX, Concord, ON, Canada). Trapping and desalting were performed at a flow rate of 4 μL/min for 5 min, with 100% solvent A (water/acetonitrile/formic acid (98/2/0.1%; B, 2/98/0.1%)). Then, an elution gradient of 8–38% solvent B was used on an analytical column (75 μm × 15 cm C18–3 μm 120 Å; ChromXP, Eksigent) for 30 min. Information-dependent acquisition (IDA) MS techniques were used to acquire tandem MS data on a Triple TOF 6600 tandem mass spectrometer (SCIEX), fitted with a Nanospray III ion source. Data were acquired using an ion spray voltage of 2.4 kV, curtain gas of 35 PSI, nebulizer gas of 12 PSI, and an interface heater temperature of 150˚C. The MS was performed with TOF-MS scans. For IDA, survey scans were acquired in 250 ms and up to 40 product ion scans (50 ms) were collected if a threshold of 260 cps with a charge state of 2-4 was exceeded. A rolling collision energy setting was applied to all precursor ions for collision-induced dissociation. Dynamic exclusion was set for 16s.

The MS/MS data were analyzed for protein identication and quantication using ProteinPilot Software v.5.0 (SCIEX). The local false discovery rate was estimated with the integrated PSPEP tool in the ProteinPilot Software as 1.0%, after searching against a decoy concatenated uniprot human protein database (20,191 entries). The following settings were then selected: sample type, identification; cystine alkylation, iodoacetamide; digestion, trypsin; instrument, TripleTOF 6600; species, none; search effort, thorough ID. For each identified peptide confidence should be >95%, and the proteins should have at least 2 unique peptides.

### Computer Modeling Analysis

The initial protein structure was built based on its X-ray crystal structure (PDB code: 2GVJ) (Khan et al., 2006). Computational docking was performed using the program Autodock Vina (Trott and Olson, 2010). The search space for docking was large enough to include the default pocket of the protein and for the ligand to rotate in. Potential binding configurations were then selected based on their binding affinity energy. Molecular dynamic simulations were performed from the selected docking conformation with Amber18 software using FF14SB force filed for the protein, GaFF2 forcefield for the ligand, and the TIP3P water model (Jorgensen et al., 1983; Maier et al., 2015). The protein was solvated in a rhombic octahedral box with periodic boundary conditions and a distance of 10 Å between the boundary and the nearest protein atoms. Sodium and chloride ions were added to neutralize the simulated system. The system was minimized for 10,000 steps using a steepest descent algorithm, followed by a 1 ns heating process to increase the temperature from 10 to 310 K, and 1 ns of NPT simulation with weak restraints on heavy atoms. The 20 ns of NPT MD production simulation was performed at 310 K, and snapshots from the last 10 ns were used for MM/GBSA calculations.

### *In Vitro* Nicotinamide Phosphoribosyltransferase Inhibition Assays

Dezocine (40 μg/ml), 10 nM FK866 and 20 μM NMN in ddH_2_O were prepared, and *in vitro* NAMPT enzyme inhibitory activity assays were performed using the NAMPT Colorimetric Assay kit (Abcam, Cambridge, United Kingdom), according to manufacturers’ protocol.

### Determination of Intracellular NAD^+^ Levels

MDA-MB-231 and BT549 cells, cultured in 6-well plates, were treated with dezocine at 0, 10, 20, 40 ug/ml or 10 nM FK866 for 48 h. Cells were then collected using a NAD^+^/NADH extraction buffer. Intracellular NAD^+^ content was determined with a NAD^+^/NADH Assay kit with WST-8 (Beyotime Institute of Biotechnology, Beijing, China), according to the manufacturer’s protocol. The resultant value was normalized to total cell number.

### Knockdown of Nicotinamide Phosphoribosyltransferase

NAMPT-knockdown cells were generated through lentiviral-mediated delivery of NAMPT small hairpin (sh)RNA. The shRNA oligos were synthesized by GENEWIZ (South Plainfield, NJ, United States) and cloned into the pLKO.1 expression construct (using pLKO.1-scramble shRNA as control). The shRNA sequences used were provided in Supplementary Table S3. The resultant pLKO.1-shRNA plasmids were co-transfected into HEK293T cells with the pCMV-VSV-G packaging plasmids and envelope pCMV-delta-8.2 envelope plasmids for production of the shRNA lentivirus, as described below. After 48 h, the supernatant fractions from the cell cultures were collected and filtered through a 0.45 μm filter. Cells were then infected with the viral supernatant fractions and supplemented with polybrene. The culture medium was replaced with fresh growth medium 16 h post-infection, with 2 μg/ml puromycin for 48 h-selection. The cells were cultured in this medium until the control cells died. Knockdown efficiency was then evaluated by qPCR and western blot analysis. The shRNA sequences used are listed in Supplementary Table S3.

### Transient Transfection

The MOR and KOR expression plasmids, OPRM1-Tango and OPRK1-Tango, were obtained from Addgene (Watertown, MA, United States). A total of 5 × 105 MDA-MB-231 or BT549 cells were inoculated in a 100-mm dish. When the cells reached 70% confluence, they were transfected with the target plasmids and Lipofectamine 3000™ (Life Technologies; Thermo Fisher Scientific, Inc.) and configured in Opti-MEM for 10 min at room temperature. The transfection mixture was incubated with TNBC cell lines for 48 h, and protein and gene expression were detected by western blot or qPCR, respectively.

### Xenografts in Nude Mice

All animal research procedures were performed according to the protocols of the Animal Care and Use Ethics Committee of Shenzhen University Health Science Center and all animals were treated in strict accordance with protocols approved by the Institutional Animal Use Committee of the Health Science Center, Shenzhen University. Female NU/NU mice (Charles River. Beijing, China, ∼4–6 weeks old; *n* = 10) were subcutaneously injected with 10X10^6^ MDA-MB-231 cells in 200 uL PBS supplied with 25% of Matrigel (Corning, NY, United States) on the right side of the back. 3 weeks after inoculation, these mice were then randomly allocated into control group (*n* = 5) and experimental groups (*n* = 5) for treatment. PBS (control group) or dezocine (30 mg/kg; experimental group) was daily intraperitoneally injected for 4 weeks. The tumor growth was monitored by measurement of tumor diameter twice a week, and the tumor volume was calculated with the formula: volume = (length x width^2^)/2. At the end of treatment, the mice were sacrificed and tumor xenografts were excised and weighted.

### Statistical Analysis

All experiments were performed at least in triplicate. Data were analyzed using GraphPad Prism 7 statistical software (GraphPad Software, Inc., La Jolla, CA, United States). Data are presented as the mean ± standard deviation. Two-tailed, unpaired Student’s t-tests were used to compare the difference between two groups with similar variance. For all tests, *P* < 0.05 was considered to indicate a statistically significant difference.

## Results

### Dezocine Exerts Anti-Tumor Effects on Triple-Negative Breast Cancer Cancer Cells

**FIGURE 1:**
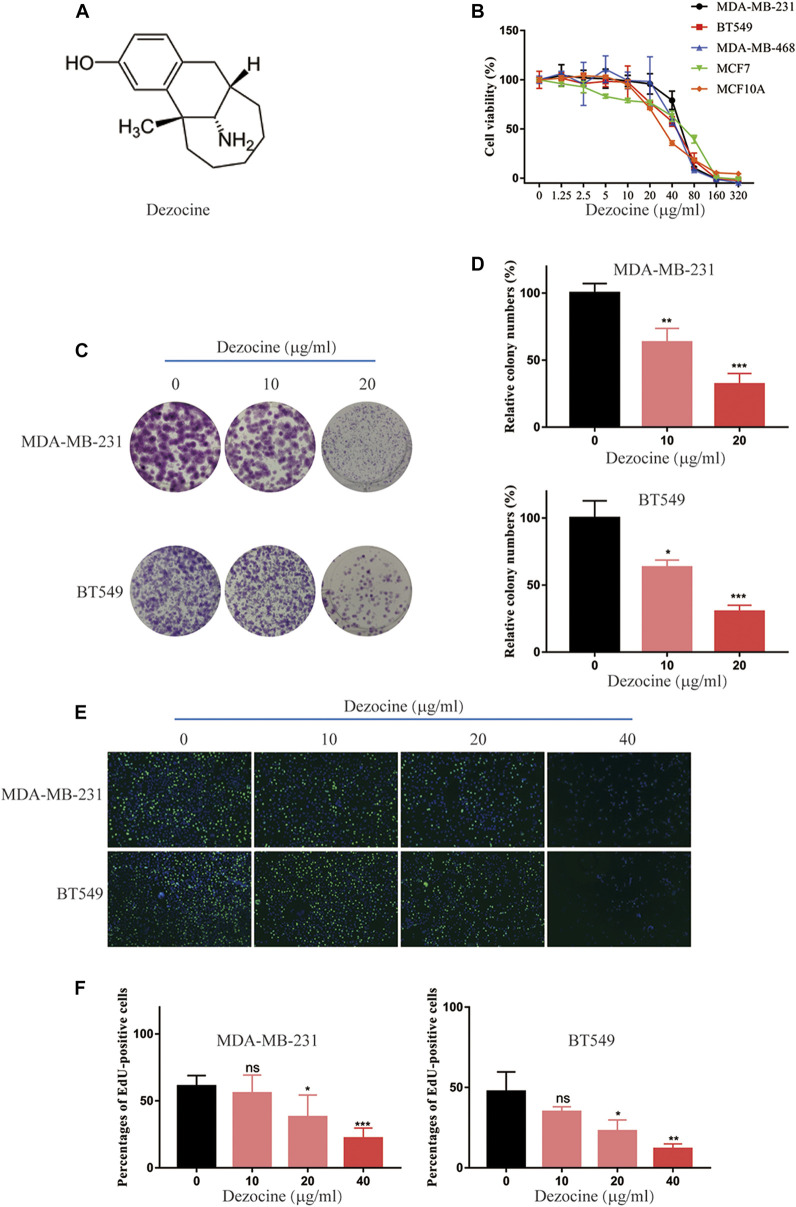
Dezocine inhibits breast cancer cell viability, colony formation and DNA synthesis. **(A)** The molecular structure of dezocine. **(B)** MDA-MB-231, BT549, MDA-MB-468, and MCF7 cells were treated with the indicated concentrations of dezocine for 48 h, and cell viability assays were performed. **(C)** Representative images of colony formation assays performed with dezocine-treated MDA-MB-231 and BT549 cells, with **(D)** quantification. **(E)** DNA synthesis was measured in dezocine-treated MDA-MB-231 and BT549 cells using EdU incorporation assays, with **(F)** quantification. Percentages of EdU-positive cells out of total cells are shown. *P < 0.05, **P < 0.01 and ***P < 0.001 vs. negative control.

Dezocine (Figure 1A) is an opioid analgesic used to relieve pain in cancer patients, but its direct influence on cancer cells remains to be determined. In the present study, we initially tested the effect of dezocine treatment on cell viability in a range of cell lines, which included TNBC, ER-positive breast cancer and mammary epithelial cell lines. The viability of all the cell lines was inhibited in a dose-dependent manner by treatment with dezocine for 48 h (Figure 1B). The IC50 values for all cell lines was in the 30–50 μg/ml range, suggesting that dezocine does not exhibit cell type specificity. Immortalized MCF10A mammary epithelial cells were also sensitive to dezocine (IC50 = 32.12 μg/ml; Table 1). Colony formation assays further confirmed the dose-dependent, dezocine-induced inhibition of MDA-MB-231 and BT549 TNBC cell proliferation, with fewer cells visible on the plates as the dose increased from 10 μg/ml to 20 μg/ml (Figures 1C,D). Furthermore, EdU assays were conducted to analyze the impact of dezocine treatment on DNA synthesis in MDA-MB-231 and BT549 cells. Dezocine significantly decreased DNA synthesis in both MDA-MB-231 and BT549 cells in a dose-dependent manner (Figures 1E,F); consistent with the results of cell viability and clonogenic assays.

**TABLE 1:** IC50 of dezocine in TNBC, ERα^+^ breast cancer and mammary epithelial cell lines.

Types	Cell line	IC50 (μg/ml)
ERα^+^	MCF7	44.06
TNBC	MDA-MB-231	52
	MDA-MB-468	43.22
	BT549	40
Normal	MCF10A	32.12

TNBC, triple negative breast cancer; ER, estrogen receptor.

### Dezocine Induces Apoptosis in MDA-MB-231 and BT549 Cells

**FIGURE 2:**
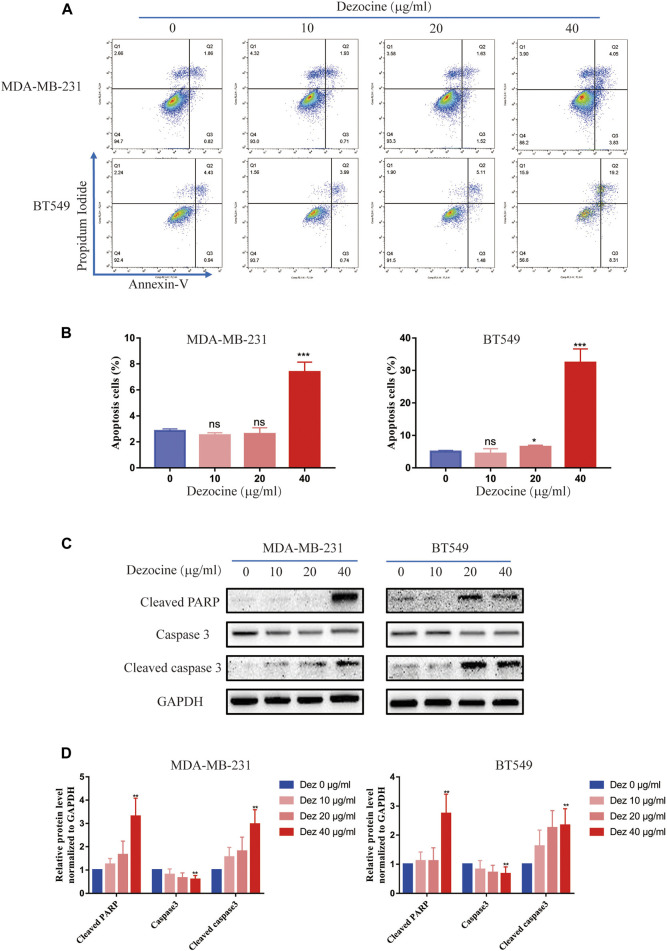
Dezocine induces apoptosis in MDA-MB-231 and BT549 cells. **(A)** Apoptosis was measured with Annexin V and PI staining in MDA-MB-231 and BT549 cells treated with dezocine for 48 h, followed by flow cytometry. **(B)** Quantification of flow cytometry data. **(C)** Apoptosis-related protein expression was detected by western blot. **(D)** Quantification of western blot data. *P < 0.05, **P < 0.01 and ***P < 0.001 vs. control.

Flow cytometry revealed that dezocine treatment (10–40 μg/ml; 48 h) significantly increased the proportion of Annexin V-positive apoptotic cells, and this effect was dose-dependent (Figures 2A,B). Furthermore, the protein expression of apoptotic markers was upregulated after dezocine treatment (Figures 2C,D), which was consistent with the flow cytometry results.

### Dezocine Inhibits Cell Migration and Invasion in MDA-MB-231 and BT549 Cells

**FIGURE 3:**
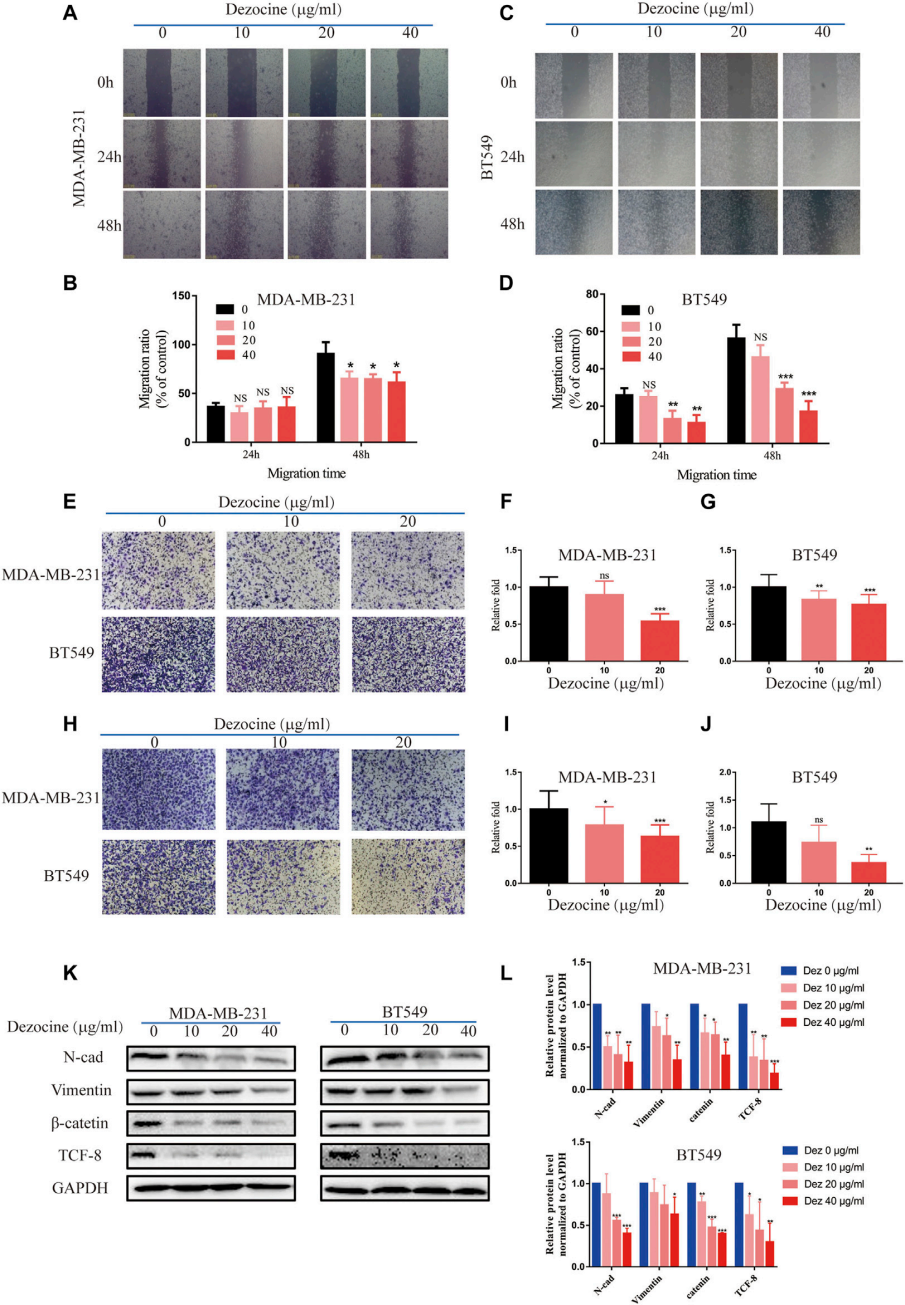
Dezocine inhibits MDA-MB-231 and BT549 cell migration and invasion. **(A)** Wound healing assays revealed that dezocine inhibited MDA-MB-231 cell migration, with **(B)** quantification; similar results were found for **(C)** BT459 cells, with **(D)** quantification. **(E)** Transwell assays were performed to detect cell migration following dezocine treatment, with quantification of **(F)** MDA-MB-231 cells and **(G)** BT549 cells. **(H)** Transwell assays with Matrigel were performed to detect cell invasion following dezocine treatment, with quantification in **(I)** MDA-MB-231 and **(J)** BT549 cells. **(K)** MDA-MB-231 and BT549 cells were treated with dezocine for 48 h, and the expression levels of epithelial and mesenchymal markers were detected by western blot. **(L)** Quantification of western blot data. *P < 0.05, **P < 0.01 and ***P < 0.001 vs. control.

The results of the wound healing assay revealed that both MDA-MB-231 and BT549 cell migration was suppressed by dezocine treatment for 24 or 48 h (Figures 3A–D). This effect was once again dose-dependent. Transwell migration assays confirmed this result, as well as its dose-dependent nature (Figures 3E–G). Similar results were also obtained from Transwell assays using Matrigel, suggesting that MDA-MB-231 and BT549 cell invasion was also inhibited by 10 or 20 ug/ml dezocine treatment (Figures 3H–J). Furthermore, protein expression of the mesenchymal markers N-cadherin, vimentin, TCF8 and beta-catenin was downregulated by dezocine treatment for 48 h (Figures 3K,L). Taken together, these results clearly demonstrate the inhibitory effect of dezocine on the migration and invasion of TNBC cell lines *in vitro*.

### Dezocine Suppresses the Progression of Triple-Negative Breast Cancer in An Opioid Receptor Independent Manner

**FIGURE 4:**
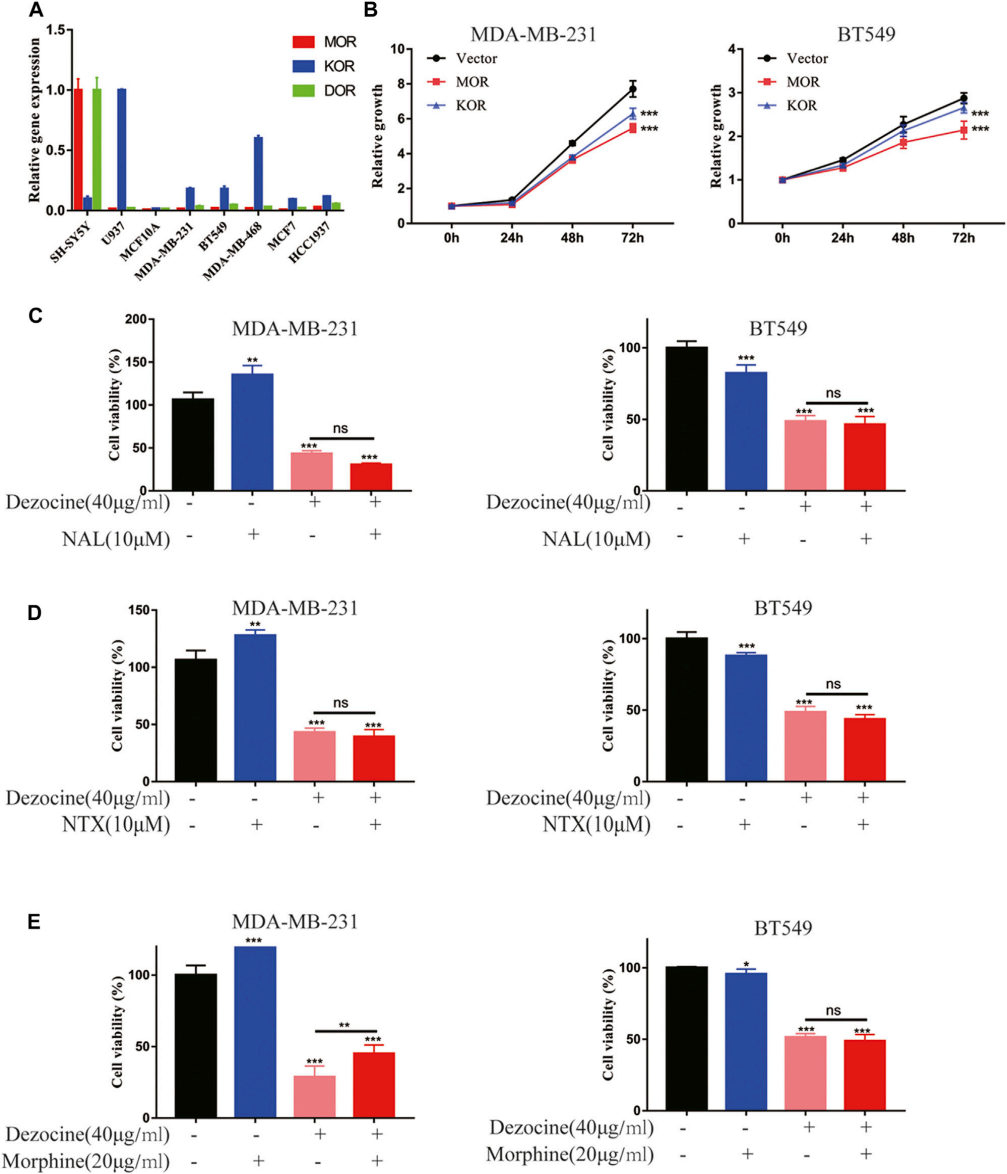
Dezocine suppresses TNBC progression in an opioid receptor-independent manner. **(A)** mRNA expression levels of the opioid receptors MOR, KOR and DOR were determined in the indicated cell lines, including breast cancer cells and MCF10A breast epithelial cells, with SH-SY5Y and U937 as controls. **(B)** MDA-MB-231 and BT549 cells were transiently transfected with MOR or KOR plasmids, and cell proliferation was determined. MDA-MB-231 and BT549 cells were pre-treated with **(C)** NAL or **(D)** NTX or **(E)** morphine for 15 min prior to treatment with dezocine for 48 h, and cell viability was measured with a CCK8 assay. *P < 0.05, **P < 0.01 and ***P < 0.001 vs. control.

Dezocine functions as an analgesic via opioid receptors, but whether the mechanism underlying the dezocine-induced inhibition TNBC occurs via these receptors remains unknown. We first detected the mRNA expression of opioid receptors (MOR, KOR and DOR) with RT-qPCR in breast cancer cell lines and normal MCF10A breast epithelial cells, using the SH-SY5Y neuroblastoma cell line and U937 monocyte cell line as controls. The mRNA expression levels of opioid receptors were comparatively low in both normal breast and breast cancer cell lines, with MOR expression levels being particularly reduced (Figure 4A). Furthermore, we tested the effect of opioid receptor overexpression in TNBC cells, and found that MOR and KOR overexpression inhibited MDA-MB-231 and BT549 cell proliferation (Figure 4B). To further investigate whether dezocine works as an opioid receptor agonist here, MDA-MB-231 and BT549 cells were incubated with 40 μg/ml dezocine for 48 h after pretreatment for 15 min with 10 uM NAL/NTX, which are opioid receptor antagonists. However, neither NAL nor NTX rescued MDA-MB-231 and BT549 cell viability following dezocine treatment (Figures 4C,D). Considering dezocine also acts as an antagonist of opioid receptors, TNBC cells were treated with 40 μg/ml dezocine for 48 h after pretreatment for 15 min with 20 ug/ml morphine, which acts as an agonist of opioid receptors. The inhibitory effect of dezocine on cell viability was not reversed by morphine (Figure 4E), and these data together implied that dezocine exerts its anti-tumor effect on TNBC cells in an opioid receptor-independent manner.

### Dezocine Inhibits Nicotinamide Phosphoribosyltransferase Enzyme Activity and Reduces Cellular NAD Content Through Targeting Nicotinamide Phosphoribosyltransferase

**FIGURE 5:**
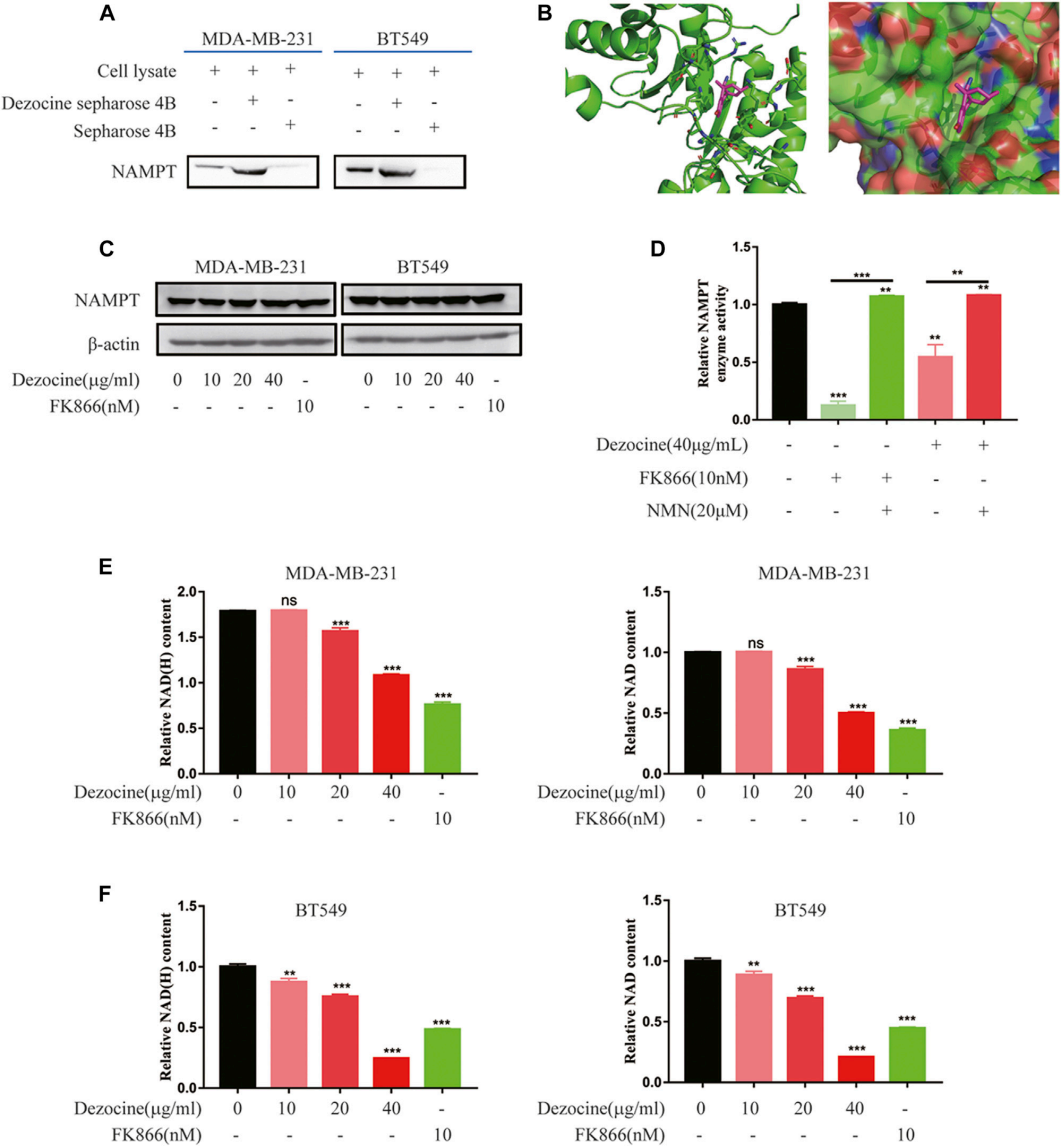
Dezocine inhibits NAMPT enzyme activity, which reduces cellular NAD content. **(A)** A pull-down assay with dezocine-sepharose 4B beads was performed in MDA-MB-231 and BT549 cells, and NAMPT protein expression was detected with western blot analysis. **(B)** Computer modeling analysis revealed that dezocine binds to NAMPT. NAMPT is presented as a graphic, with the residues nearby to dezocine represented as sticks in the left. NAMPT is presented as the surface in the right. **(C)** MDA-MB-231 and BT549 cells were treated with dezocine for 48 h, and NAMPT expression levels were detected. FK866 was used as the control. **(D)** NAMPT enzyme activity was examined *in vitro*. NAD content was then detected in **(E)** MDA-MB-231 and **(F)** BT549 cells. **P < 0.01 and ***P < 0.001 vs. control, with additional comparisons indicated by lines.

To reveal the direct target and molecular mechanisms underlying the effects of dezocine, a pull-down assay and HPLC-MS/MS were performed. The proteins pulled down from MDA-MB-231 lysate by dezocine-sepharose 4B beads were separated by SDS-PAGE, and LC-MS/MS was performed to compare them with those pulled down by sepharose 4B beads (Supplementary Figure 1A). Among these candidate targets from LC-MS/MS (Supplementary Table S3), NAMPT was confirmed to be captured by dezocine-conjugated beads in both MDA-MB-231and BT549 cells through western blot analysis, suggesting that dezocine targeted NAMPT directly (Figure 5A). Furthermore, computer modeling analysis indicated that dezocine binds in the NAMPT pocket, and the free energy of dezocine kinetically bound into this pocket was −17.4 kcal/mol (Figure 5B). This data further confirmed that NAMPT is the direct target of dezocine.

Next, NAMPT mRNA expression was confirmed in breast cancer cell lines by RT-qPCR, and higher NAMPT expression levels were observed in MDA-MB-231 and BT549 cell lines (Supplementary Figure 2A). However, following dezocine treatment for 48 h, NAMPT protein expression did not change in MDA-MB-231 and BT549 cells (Figure 5C). This indicated that dezocine did not affect NAMPT protein stability.

NAMPT is a rate-limiting enzyme involved in the nicotinamide adenine dinucleotide salvage pathway. To determine the activity of the enzyme with dezocine binding, an NAMPT activity assay was performed and the NAMPT inhibitor FK866 was used as a control. Both dezocine and FK866 inhibited NAMPT enzyme activity, while NMN rescued NAMPT activity *in vitro* (Figure 5D). Furthermore, as demonstrated with an NAD/NADH assay, dezocine reduced NAD production in a dose-dependent manner in both MDA-MB-231and BT549 cells (Figures 5E,F). Together, these results suggested that dezocine bound directly to NAMPT and inhibited the activity of the enzyme.

FK866T is an NAMPT inhibitor that reduces cell proliferation by decreasing intracellular NAD levels. In TNBC cells, the inhibitory effects of FK866 were confirmed by cell viability assays, while dezocine showed a similar inhibitory effect (Supplementary Figures 2B,C). The suppression of cell growth was further confirmed by knockdown of NAMPT (knockdown efficiency was detected, Supplementary Figure 2D) in MDA-MB-231and BT549 cells (Supplementary Figures 2E,F).

### Dezocine Inhibits Triple-Negative Breast Cancer Tumor Growth *In Vivo*

**FIGURE 6:**
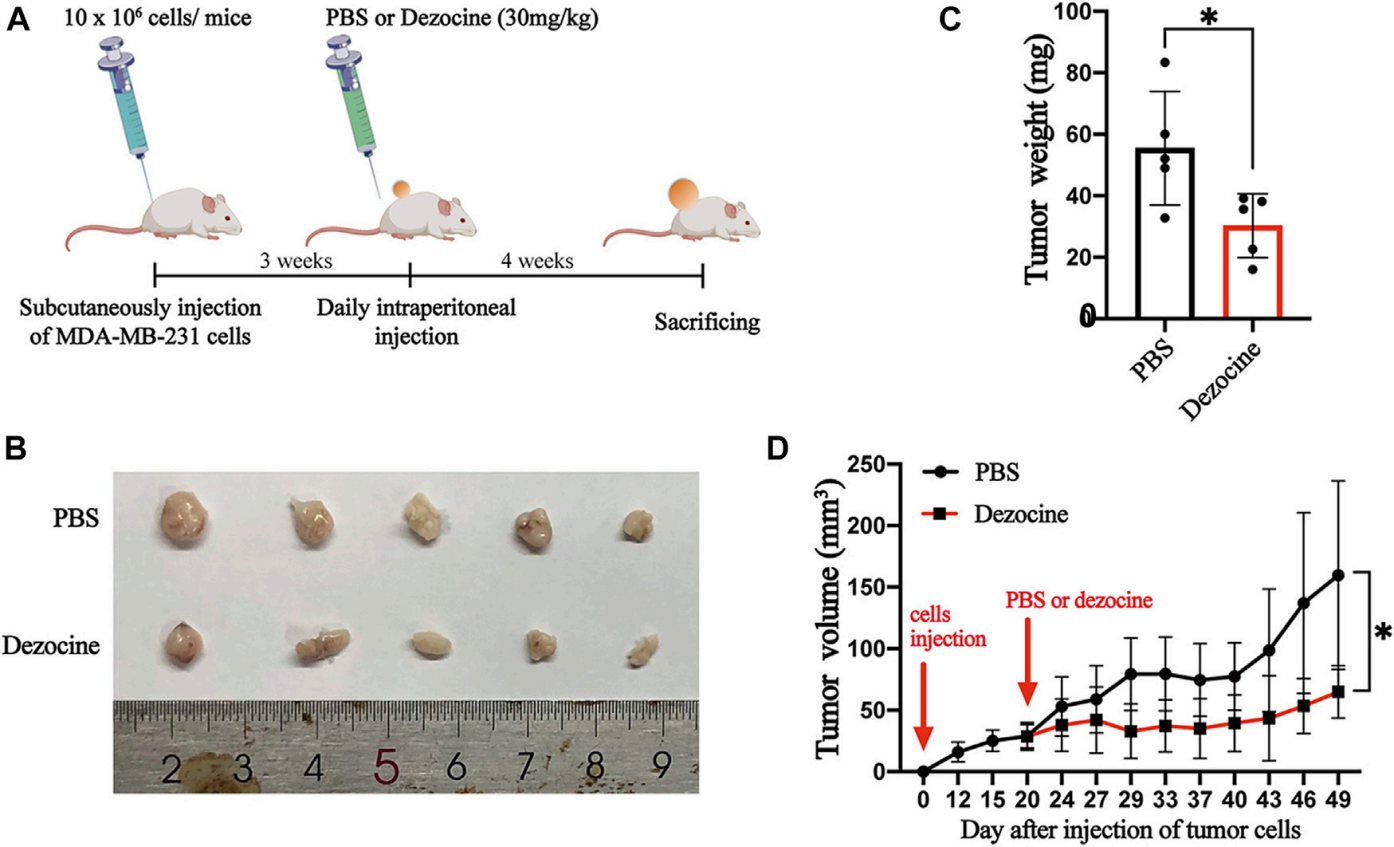
Dezocine exhibits anti-tumor effects on xenografts *in vivo*. **(A)** MDA-MB-231 cells (10 × 10^6^ cells) was subcutaneously injected into the flank of mice. 3 weeks after tumor cells inoculation, a total of 30 mg/kg dezocine in PBS solution was daily injected intraperitoneally for 4 weeks. **(B)** The photographs of tumors from mice treated with PBS or dezocine. **(C)** The mean of tumor weights was measured after PBS or dezocine treatment (*p* = 0.03). **(D)** The tumor volumes from mice were measured twice a week post-injection (*p* = 0.02). *P < 0.05 vs. control.

Having shown the effects of dezocine *in vitro*, we subsequently wanted to determine the inhibitory effects of dezocine *in vivo*. We subcutaneously injected MDA-MB-231 cells (10 × 10^6^) into the right flanks of nude mice. 3 weeks after inoculation, the mice were exposed to PBS or dezocine (30 mg/kg) treatment for 4 weeks (Figure 6A). Xenografts taken from mice in the dezocine-treated group were significantly smaller than those taken from the PBS-treated group (Figures 6B,C). Indeed, we measured the tumor volume twice a week after injection of MDA-MB-231 cells, and found that it was significantly reduced in the dezocine-treated group compared to the PBS-treated group (Figure 6D). However, there was no significant difference in the body weights between the two groups of mice (Supplementary Figure S3), suggesting the dosage of dezocine we used was systemic safety.

## Discussion

Pain remains a prevalent concern for patients with cancer, and has a significant impact on clinical outcome. Opioids are widely used as clinical analgesic agents for cancer patients. Dezocine is a synthetized opioid analgesic and is broadly prescribed for pain relieving in China, occupying over 45% of the domestic market of opioid analgesics. Thus, it is very important to understand the role of dezocine in cancer treatment. However, the effect of dezocine on cancer cells remains unknown. In the present study, we demonstrated that dezocine inhibited DNA synthesis, cell proliferation, cell migration and invasion in TNBC cell lines *in vitro*. Dezocine treatment also induced apoptosis in TNBC cells, confirmed by the upregulation of pro-apoptotic proteins, such as cleaved PARP and cleaved caspase 3. Importantly, our xenograft models demonstrated the inhibitory effects of dezocine treatment on TNBC tumor growth *in vivo*. These results clearly demonstrate the anticancer effects of dezocine in TNBC.

Dezocine is a MOR and KOR mixed agonist-antagonist, with a higher affinity for MOR (Liu et al., 2014; Wang et al., 2018). MOR overexpression has been observed in human NSCLC, prostate cancer and HCC, and is regarded as a molecular marker for poor prognosis. For example, MOR silencing and MOR antagonists treatment have previously been demonstrated to suppress carcinoma progression (Mathew et al., 2011; Zylla et al., 2013; Chen et al., 2019; Li et al., 2019). However, the results of our study indicated that opioid receptor expression in breast cancer cell lines and mammary epithelial cell lines is relatively low compared with SH-SY5Y and U937 cells. This suggested that the expression pattern of opioid receptors in breast cancer is completely different from that in lung cancer and HCC. When MOR and KOR were overexpressed in TNBC cells, cell proliferation was suppressed; but this is not consistent with the results seen in lung cancer and HCC. Furthermore, neither NAL/NTX, which acts as an antagonist, nor the agonist morphine, managed to suppress the inhibitory effects of dezocine. This indicated that dezocine acts in an opioid receptor independent manner, suggesting that opioid receptor function depends on cancer type and tissue specificity.

Our pull-down assay followed by LC-MS/MS found that dezocine targeted NAMPT directly; and computer modeling analysis confirmed that dezocine bound to NAMPT. NAMPT, also known as pre-B-cell colony-enhancing factor 1 (PBEF1) or visfatin, acts as a rate-limiting enzyme in the nicotinamide adenine dinucleotide (NAD^+^) salvage pathway. It catalyzes the condensation of nicotinamide and 5-phosphoribosyl-1-pyrophosphate to nicotinamide mononucleotide during NAD^+^ biosynthesis, and extracellular NAMPT exerts additional cytokine-like activity (Garten et al., 2015). NAD is a co-enzyme that participates in a number of cell metabolic pathways, including glycolysis, with increased NAD levels enhancing glycolysis. This can provide cancer cells with energy and promote tumor progression. Furthermore, NAD is a substrate of NAD-dependent enzymes such as poly (ADP-ribose)polymerase (PARP), sirtuins, and NAD glycohydrolase, which regulate DNA repair, gene expression and stress responses, all of which have implications for cancer (Yaku et al., 2018). Thus, through its control of NAD biosynthesis, NAMPT has a crucial role in cancer cell metabolism. NAMPT overexpression has been observed in multiple malignant tumors, including breast, ovarian, thyroid, gastric, prostate and colorectal cancers, gliomas, and malignant lymphomas (Tan et al., 2015). NAMPT promotes the proliferation and survival of rapidly dividing cancer cells by elevating NAD levels and enhancing glycolysis (Tan et al., 2013; Yamamoto et al., 2017). As a result, NAMPT is considered a promising novel therapeutic target for cancer treatment. NAMPT inhibitors such as FK866, GMX1777, and GMX1778, have been developed as a candidate novel therapeutic strategy for cancer (Yaku et al., 2018). In our study, NAMPT expression was verified in breast cancer cell lines and mammary epithelial cell lines, and the results were in accordance with those previously obtained in a range of solid tumors, including breast cancer (Shackelford et al., 2013). A study including 176 breast cancer patients also demonstrated that higher NAMPT levels are correlated with poorer survival, with high-grade tumors having significantly higher NAMPT/p73 mRNA ratios (Sharif et al., 2016). Furthermore, NAMPT has been found to induce breast cancer cell proliferation through the AKT/PI3K and ERK/MAPK signaling pathways, and to protect against apoptosis (Gholinejad et al., 2017). In the present study, MDA-MB-231and BT549 cell proliferation was suppressed by NAMPT knockdown, which are similar to those results found previously.

Furthermore, we found that dezocine treatment led to the inhibition of NAMPT activity and the reduction of cellular NAD, and this effect was dose-dependent. As a result, TNBC cell proliferation was suppressed. Similar results were observed following FK866 treatment in our study. FK866 was the first inhibitor of NAMPT to be developed, and is regarded as a candidate novel therapeutic drug through blocking NAMPT activity. FK866 induced apoptosis-mediated cell death in chronic lymphocytic leukemia cells and HepG2 liver carcinoma cells (Hasmann and Schemainda, 2003; Gehrke et al., 2014), inhibited the epithelial-mesenchymal transition in hepatocarcinoma MHCC97-H cells (Zhang et al., 2018). However, NAMPT has also been reported to induce the epithelial-to-mesenchymal transition independently of its enzymatic activity (Soncini et al., 2014). Furthermore, NAMPT knockdown has been found to increase the aggressiveness of human breast cancer metastasis through the regulation of integrins (Santidrian et al., 2014). The results of the present study suggested that dezocine suppresses cell migration and invasion in TNBC cells, however, the underlying molecular mechanisms require to be further elucidated.

In summary, the present study is the first to report the efficacy of dezocine against TNBC *in vitro* and *in vivo.* Furthermore, we demonstrated that dezocine binds directly to NAMPT, inhibiting its enzyme activity and downregulating NAD in TNBC cells. The lack of effective treatments for TNBC is a global health concern, and the development novel treatment strategies is urgently required. Drug repurposing has emerged as a novel strategy for cancer therapy. Dezocine represents a potential candidate treatment for TNBC and perhaps other cancers, and, furthermore, NAMPT may represent a candidate therapeutic target in TNBC. Notably, previous study has shown that the combination of NAMPT inhibitor FK866 with olaparib inhibited TNBC growth *in vivo* than either single agent alone (Bajrami et al., 2012), supporting the potential use of dezocine alongside Olaparib or other therapeutic agents, to increase overall efficacy in TNBC. The further investigation is warranted in future studies.

## Data Availability

The original contributions presented in the study are publicly available. This data can be found here: dezocine pull down LC-MSMS in MDA-MB-231 CELLS has been deposited in the ProteomeXchange repository, accession number: PXD022583.
