# Supplementary material for: Dezocine, An Opioid Analgesic, Exerts Antitumor Effects in Triple-Negative Breast Cancer by Targeting Nicotinamide Phosphoribosyltransferase
Source: Front Pharmacol. 2021 Apr 12;12:600296. doi: 10.3389/fphar.2021.600296 (PMC8072669; doi:10.3389/fphar.2021.600296)
Supplement: Supplementary file 1 [file datasheet1.pdf]

Supplementary Data and Materials

Supplementary Figures

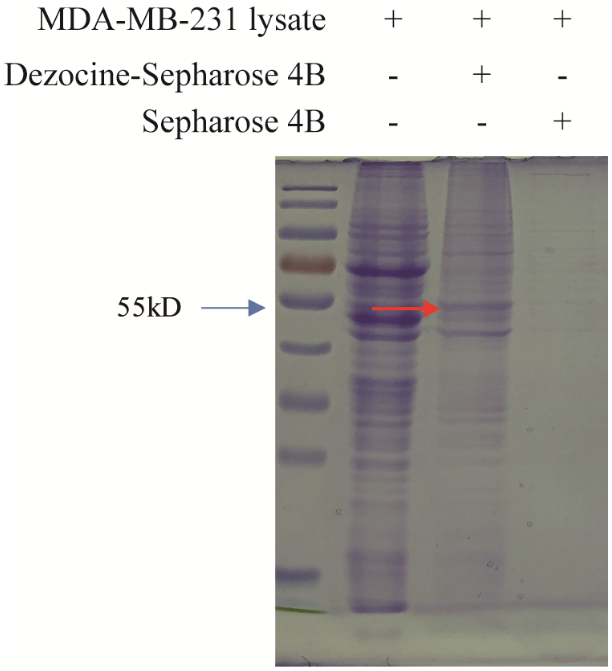

Supplementary Figure 1. SDS-PAGE gel indicating proteins from MDA-MB-231 lysate that were pulled down by dezocine-sepharose 4B beads. The PAGE gel was stained with Coomassie Blue. The differential bands labelled by red arrows were subjected to LC-MS/MS.

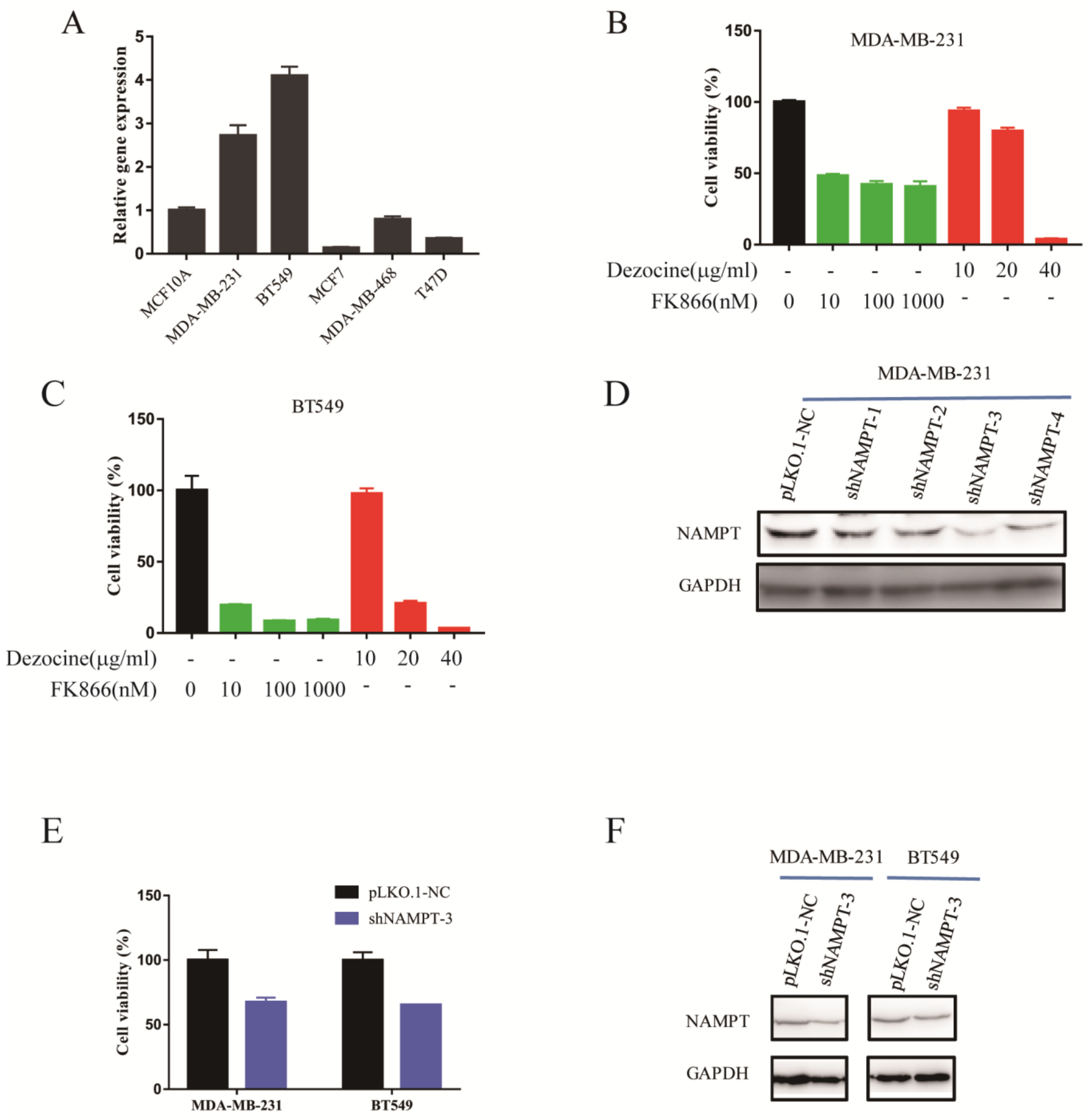

Supplementary Figure 2. Cell proliferation was suppressed by transient knockdown of NAMPT or FK866 treatment in MDA-MB-231 and BT549 cells. (**A)** NAMPT mRNA expression was detected in breast cancer cell lines and MCF10A cells by RT-qPCR. **(B)** MDA-MB-231 cells and **(C)** BT549 cells were treated with FK866 or dezocine for 72 h, and cell viability was measured with a CCK-8 assay. (**D)** The transient knockdown efficiency of NAMPT in MDA-MB-231 cells was confirmed by western blot analysis. (**E)** Transient knockdown of NAMPT resulted in decreased MDA-MB-231and BT549 cell viability. (**F)** Transient knockdown efficiency was confirmed by western blot analysis.

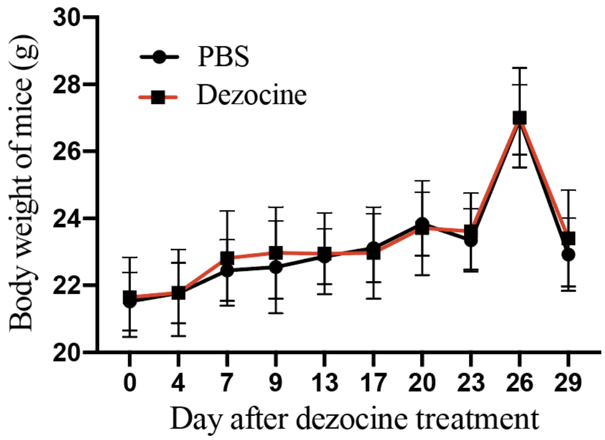

Supplementary Figure 3. Body weight of nude mice in dezocine-treated and control groups.

Body weight was measured twice a week after injection of tumor cells. There was no significant difference between the dezocine -treated and control groups.

Supplementary Table 1. Antibodies used for western blot analysis.

Name	MW (kDa)	Source	Company	Dilution	Cat. No.
PARP	89	Rabbit	Cell Signaling Technology	1:1000	9542
Caspase 3	35	Rabbit	Cell Signaling Technology	1:1000	9665
Cleaved Caspase 3	17/19	Rabbit	Cell Signaling Technology	1:1000	9664
N-cadherin	140	Rabbit	Cell Signaling Technology	1:1000	13116
vimentin	57	Rabbit	Cell Signaling Technology	1:1000	5741
β-catenin	92	Rabbit	Cell Signaling Technology	1:1000	8480

TCF-8	200	Rabbit	Cell Signaling Technology	1:1000	3396
NAMPT	54	Rabbit	Abcam	1:1000	Ab58640
GAPDH	37	Mouse	Abmart	1:5000	M2006M
Anti-Mouse IgG	/	Goat	Abcam	1:5000	Ab97023
Anti-Rabbit IgG	/	Goat	Abcam	1:5000	Ab97051

Supplementary Table 2. Primer sequences used for qPCR.

Primers	Sequence (5'-3')	Company
MOR-forward	CTCTCTGGCTCCAAAGAAAAGGA	GENEWIZ
MOR-reverse	CAATGCAGAAGTGCCAAGAAACA	GENEWIZ
KOR-forward	GCTGCTCTCTCCAGCTATTACTTCT	GENEWIZ
KOR-reverse	GCAGGATCCTGAACTGTATTTCGGACTC	GENEWIZ
DOR-forward	GGCTACGCCAATAGCAGCCTCAAC	GENEWIZ
DOR-reverse	ACCGCCGGGACCATCGGACGGG	GENEWIZ
GAPDH-forward	AGGTGAAGGTCGGAGTCAAC	GENEWIZ
GAPDH -reverse	AGTTGAGGTCAATGAAGGGG	GENEWIZ
NAMPT-forward	GCCAGCAGGGAATTTTGTTA	GENEWIZ

NAMPT -reverse	GATGTGCTGCTTCCAGTTCA	GENEWIZ

Supplementary Table 3. Partial analysis results of LC-MS/MS.

Name	Peptides (95%)
Fatty acid synthase OS=Homo sapiens GN=FASN PE=1 SV=3	26
**Nicotinamide phosphoribosyltransferase OS=Homo sapiens GN=NAMPT PE=1 SV=1**	**27**
Eukaryotic initiation factor 4A-I OS=Homo sapiens GN=EIF4A1 PE=1 SV=1	34
26S protease regulatory subunit 6A OS=Homo sapiens GN=PSMC3 PE=1 SV=3	25
Elongation factor 1-gamma OS=Homo sapiens GN=EEF1G PE=1 SV=3	22
DnaJ homolog subfamily A member 1 OS=Homo sapiens GN=DNAJA1 PE=1 SV=2	27
Heat shock protein HSP 90-beta OS=Homo sapiens GN=HSP90AB1 PE=1 SV=4	21
Alpha-enolase OS=Homo sapiens GN=ENO1 PE=1 SV=2	25
26S protease regulatory subunit 8 OS=Homo sapiens GN=PSMC5 PE=1 SV=1	24
Eukaryotic translation initiation factor 3 subunit E OS=Homo sapiens GN=EIF3E PE=1 SV=1	28
Pyruvate kinase PKM OS=Homo sapiens GN=PKM PE=1 SV=4	23
Polyadenylate-binding protein 1 OS=Homo sapiens GN=PABPC1 PE=1 SV=2	23

Nuclease-sensitive element-binding protein 1 OS=Homo sapiens GN=YBX1 PE=1 SV=3	21
Bifunctional glutamate/proline--tRNA ligase OS=Homo sapiens GN=EPRS PE=1 SV=5	19
Alpha-actinin-1 OS=Homo sapiens GN=ACTN1 PE=1 SV=2	19
Serpin H1 OS=Homo sapiens GN=SERPINH1 PE=1 SV=2	23
EH domain-containing protein 1 OS=Homo sapiens GN=EHD1 PE=1 SV=2	19
Adenylosuccinate synthetase isozyme 2 OS=Homo sapiens GN=ADSS PE=1 SV=3	17
Tubulin gamma-1 chain OS=Homo sapiens GN=TUBG1 PE=1 SV=2	21
RuvB-like 2 OS=Homo sapiens GN=RUVBL2 PE=1 SV=3	17

Supplementary Table 4. Sequences used for construction of shRNA.

Name	Sequence (5'-3')
shNAMPT-1-Top	CCGGCCACCTTATCTTAGAGTTATTCTCGAGAATAACTCTAAGATAAGGTGGTTTTTG
shNAMPT-1-Bottom	AATTCAAAAACCACCTTATCTTAGAGTTATTCTCGAGAATAACTCTAAGATAAGGTGG
shNAMPT-2-Top	CCGGGTAACTTAGATGGTCTGGAATCTCGAGATTCCAGACCATCTAAGTTACTTTTTG
shNAMPT-2-Bottom	AATTCAAAAAGTAACTTAGATGGTCTGGAATCTCGAGATTCCAGACCATCTAAGTTAC
shNAMPT-3-Top	CCGGGGAATATGGTCAGGATCTTCTCTCGAGAGAAGATCCTGACCATATTCCTTTTTG
shNAMPT-3-Bottom	AATTCAAAAAGGAATATGGTCAGGATCTTCTCTCGAGAGAAGATCCTGACCATATTCC

shNAMPT-4-Top	CCGGGCTGAATATTGAACTGGAAGCCTCGAGGCTTCCAGTTCAATATTCAGCTTTTTG
shNAMPT-4-Bottom	AATTCAAAAAGCTGAATATTGAACTGGAAGCCTCGAGGCTTCCAGTTCAATATTCAGC
scramble-Top	CCGGGCGCGATAGCGCTAATAATTTCTCGAGAAATTATTAGCGCTATCGCGCTTTTTG
scramble-Bottom	AATTCAAAAAGCGCGCTAGCGCTAATAATTTCTCGAGAAATTATTAGCGCTATCGCGC
